# The Implementation of a Blended In-Person and Online Family-Based Childhood Obesity Management Program: A Process Evaluation Pilot Study

**DOI:** 10.3390/ijerph22101568

**Published:** 2025-10-15

**Authors:** Bianca DeSilva, Anna Sui, Sam Liu, Patti-Jean (PJ) Naylor

**Affiliations:** School of Exercise Science, Physical and Health Education, University of Victoria, Victoria, BC V8W 2Y2, Canada

**Keywords:** childhood obesity, process evaluation, implementation, British Columbia, Canada

## Abstract

Background: The Early Intervention Program (EIP) was a 10-week family-based healthy living intervention for children with a BMI-for-age ≥85th percentile. The effectiveness of the EIP has been previously demonstrated; however, its implementation has not been fully described. Process evaluations provide valuable insight into implementation and improve ongoing intervention delivery. Objective: The aim was to evaluate recruitment, intervention content, delivery, and implementation for quality improvement and to inform potential scale-up. Methods: A mixed-methods process evaluation design was used and represented one component of a Type I hybrid effectiveness trial. Results: The EIP reached diverse ethnic, educational, and socioeconomic backgrounds (*n* = 47). Participation barriers were transportation, scheduling, and illness. Participation facilitators were the free cost and family recreation pass, sibling inclusion, and location. Program acceptability/satisfaction was rated over 4/5 for all measures. Implementation barriers were recruitment, small group size, attendance, and limited time to deliver material. Implementation facilitators were high compatibility and feasibility, as well as the provided resources. Staff interviews showed high acceptability/satisfaction across all sites. Conclusion: The EIP was highly acceptable and feasible for families and delivery partners, but recruitment, attendance, and online engagement were implementation challenges. Program adjustments are recommended prior to scale-up. These strengths and limitations can help to inform other multi-site childhood healthy living interventions.

## 1. Introduction

Children who are overweight (BMI 85–95th percentile for age and sex) or obese (≥95th percentile for age and sex) are at an increased risk for developing a variety of adverse physical and psychological issues [1,2]. The prevalence of childhood overweight and obesity has steadily increased in recent decades in nearly all countries [3,4]. The prevalence of overweight and obesity among children and youth (ages 5 to 17) is about 30% in Canada [5]. Physical inactivity, an unhealthy diet, and increased screen time are significant contributing factors to the rise in prevalence of childhood obesity [6,7].

Family-centered childhood obesity lifestyle programs can be an effective strategy to prevent childhood obesity [8,9,10,11]. With rapid advancements and adoption of web-based technology, blended in-person and web-based program delivery formats have the potential to further improve program efficacy, feasibility, satisfaction, scalability, and flexibility [12]. Advantages of incorporating *web-based intervention* with in-person programming include flexibility for families to work at their own pace, additional contact hours, and reinforcement of the behavioural change skills learned during in-person sessions and, thus, increased adherence [13]. Overall, programs with blended delivery formats have the potential to maximise efficacy and scalability while reducing the cost compared to programs that are delivered entirely in person [14].

The Early Intervention Program (EIP: later rebranded as Generation Health in 2019) was developed as a prototype family-based early intervention childhood healthy weight program. The EIP was tailored to the needs of families in the province of British Columbia, Canada, and aligned with previous and existing programs while addressing a gap in programming. Early intervention family-based lifestyle programs in particular have been efficacious for addressing childhood obesity [8], and the scale-up of such interventions into real-world settings is important to prevent delays in wider community access [15], ultimately leading to a greater public health benefit. Our team along with our collaborators, the British Columbia Ministry of Health and Childhood Healthy Living Foundation, developed and piloted the EIP to meet the needs of families with children aged 8–12 years old who were overweight or obese (BMI ≥ 85th percentile for age and sex) and living in British Columbia.

The EIP is a 10-week family-based lifestyle intervention program aimed at improving physical activity, dietary behaviours, mental-wellbeing, sleep hygiene, and reducing screen time. Our previous studies showed that the EIP was effective relative to a control in improving a child’s daily moderate-to-vigorous physical activity (MVPA); further, the intervention group showed significant improvements in parental support for healthy eating behavior and self-regulation for healthy eating [16,17]. Furthermore, an intervention dose–response relationship was observed. Specifically, greater engagement with both the in-person and online components of the EIP resulted in a greater child MVPA level, child physical activity confidence, parental support for child physical activity and healthy eating, family habits for physical activity and healthy eating, and parental identity for physical activity [17]. Preliminary evidence showed that the intervention was not effective in improving child BMI z-scores over the 10-week program; a longer study period may be required [16].

Currently, the process evaluation for the EIP has not been fully described. Process evaluations can provide valuable insight into the implementation process, help to determine whether program activities have been implemented as intended, and identify how to improve program delivery [18]. Although our study aligns with many aspects of implementation research, it was explicitly designed and described as a process evaluation study. The purpose of this process evaluation study was to evaluate reach, recruitment, intervention content, delivery, and implementation of the EIP to enhance quality improvement and inform decisions about potential program scale-up. We hypothesised that the EIP would demonstrate high reach and recruitment (effectively reaching our target population and representative of British Columbia’s demographics), with participants and program leaders reporting that the intervention content and delivery were feasible, acceptable, and delivered with high fidelity. Additionally, this study will identify barriers and facilitators to both participation and implementation. These findings will be used to make recommendations for program improvements and to guide decisions about potential scale-up. Ultimately, awareness of these strengths and limitations can be used to help inform other multi-site childhood healthy living interventions.

## 2. Methods

### 2.1. Study Design

This implementation study was one component of a Type I hybrid effectiveness trial [19]. This process evaluation study utilised a concurrent mixed-methods design [20], integrating qualitative and quantitative data to evaluate the reach, recruitment, intervention delivery, and implementation fidelity of the program. Both data types were given equal weighting in the analysis, meaning that neither qualitative nor quantitative data were prioritised over the other in interpreting the findings.

Key factors addressed within the process evaluation were guided by Steckler & Linnan (2002), as described by Saunders et al. [21]. Additional measures from the RE-AIM framework [22] were incorporated and aligned with the minimal implementation data set for physical activity interventions proposed by McKay et al., 2019 [23]. Process evaluation measures were completed during September 2018 to March 2019. The programs were delivered at one of the local community centers across all five health authority regions in British Columbia, Canada (Prince George: YMCA of Northern BC; Kelowna: YMCA of the Okanagan; Surrey: YMCA of Greater Vancouver; Burnaby: City of Burnaby; Greater Victoria: Westshore Parks and Recreation). Participants were recruited using social media platforms (Facebook, Instagram, and Twitter), stakeholder engagement (e.g., advertising on recreation centre websites), local newspaper advertisements, and posters in recreation centers and libraries, as well as information packages mailed to physician offices. Interested participants were asked to contact the central site by telephone to learn more about the program and to determine study eligibility. Ethics approval for this study was obtained from the University of Victoria Human Research Ethics Board (Protocol Number B.C.18-024).

### 2.2. Participants

Families who met the following inclusion criteria were recruited to participate in the EIP: (1) at least one child between the ages of 8–12 years old with a BMI ≥85th percentile for age and sex, (2) the child had no known health issues such as cardiovascular disease, mental health issues, or eating disorders, (3) had one parent, caregiver, or legal guardian that was able to attend program sessions with the child, (4) the family resided in British Columbia, (5) the family was able to commit to the full program schedule. Exclusion criteria included individuals that were unable to participate in physical activity or who were unable to communicate fluently in English.

### 2.3. EIP Description

The EIP was a 10-week family-based lifestyle program targeting improvements in physical activity, healthy eating, positive mental health (e.g., gratitude, appreciation, and mindfulness activities), sleep hygiene, and reductions in screen time. The program has been described previously by Perdew et al., 2021 [16]. Program components included a weekly 90-minute in-person group session, a weekly e-session on a Family Portal, and four additional community-based group activities. Program cycles were delivered between September and December 2018 (Cycle One) and January and March 2019 (Cycle Two). The targeted outcomes were changes in both child and parent practices, as well as changes in the home environment. In-person sessions included child-only physical activity sessions aimed at improving enjoyment, confidence, motivation, and fundamental movement skills; parent-only classroom learning and group discussions to identify barriers and strategies for promoting family healthy behaviours; and family physical and positive mental health activities. In-person sessions were delivered by a trained local staff team which included a group facilitator, a physical activity facilitator, and a program assistant. Most of the staff team were already employees at their local YMCA or municipal recreation centre and all staff attended an in-person training session which included workshops on physical activity facilitation, healthy eating, positive mental health, trauma-informed practice, weight bias, and behaviour change. The healthy eating content was delivered by a Registered Dietitian who specialises in working with children who have eating disorders and their families. The group grocery store tours during the intervention were all led by a Registered Dietitian. Some of the group facilitators were also a Registered Dietitian or nutritionist. While not a requirement, several of the trained physical activity facilitators had a degree or diploma in kinesiology or physical education. The program was theoretically guided by the Multi-Process Action Control (M-PAC) framework [24,25] which emphasises social cognitive approaches to intention formation, the adoption of action control through self-regulation, and the action control maintenance phase once a behaviour becomes habitual. The M-PAC constructs are reflected in the program curriculum with the aim of introducing concepts and supporting participants in making long-term lifestyle behaviour changes. The online Family Portal featured a weekly e-lesson to be completed by families as well as healthy recipes, parent articles, videos, and suggested healthy eating and physical activity ideas so that families could engage in an additional self-directed healthy lifestyle activity each week. The program also provided four additional community-based group activities for families to navigate existing community resources and activities. Two of these activities were the same for all program locations: a group grocery store tour led by a Registered Dietitian and an outdoor exploration game using an augmented reality mobile application called Agents of Discovery. The remaining two activities were chosen and scheduled by the program leaders at each location based on input from children and parents as well as community availability. Examples of chosen activities were nature walks, geocaching, farmer’s markets, badminton, cooking classes, and frisbee golf. The intervention also included four in-person 60-minute bi-weekly group maintenance sessions delivered after the 10-week program. However, due to low attendance and low maintenance satisfaction survey response rates, these sessions were only delivered during Cycle One and therefore not included in this analysis.

### 2.4. Study Procedure

Process evaluation data from several qualitive and quantitative sources was collected at baseline, during program delivery, and at the 10-week follow-up. Qualitative data was collected through post-program interviews and focus groups with parents, program leaders, staff managers, and Childhood Healthy Living Foundation (CHLF) staff members. In order to maintain consistency [26], the same research assistant conducted all post-program interviews and focus groups; these were conducted in person where possible, and otherwise by telephone. Quantitative data was collected from baseline demographic information, program registration forms, satisfaction surveys, attendance tracking forms, and Family Portal e-session analytics.

Parents and children engaged in the outcome evaluation were also recruited for the process evaluation. The program leaders who delivered the program, recreation centre managers involved in program oversight and administration, and CHLF central coordination and technical support staff were also recruited.

### 2.5. Measures: Family Level

*Reach and Recruitment* were determined by the family demographic information (e.g., cultural, and racial background, language(s) spoken at home, employment status, household primary earner income, education level of parents, and size of family) collected at baseline.

*Dose Received* was assessed using attendance tracking forms completed by the program leader following each weekly group session and emailed to the Project Coordinator. It also included family online lesson analytics assessed using the total minutes each family engaged with the Family Portal over the intervention.

*Satisfaction, Barriers, and Facilitators to Participation* were determined using satisfaction surveys and post-program parent interviews. All questions were purposefully designed by the program evaluation team to address the EIP program specifics and process evaluation variables. The parent satisfaction survey included 24 questions (7 using a 5-point Likert scale and 17 open-ended). These questions addressed the satisfaction with and usefulness of weekly sessions and program components, satisfaction and usefulness of online Family Portal components, usefulness and appropriateness of information provided (during sessions and in the program binder), aspects of program delivery, barriers and facilitators to participation, program impacts on their family, and areas for program improvement. The child satisfaction survey included 17 questions (10 using a 5-point Likert scale and 7 open-ended). These addressed the satisfaction with and usefulness of weekly sessions and program components, response to program leaders, changes towards living a healthy life, favorite and least favorite parts of the program, and what children would tell their friends about the program. The post-program parent interviews and focus groups were semi-structured and included 14 open-ended questions addressing the impact of the program on their family, major lessons learned, “best and worst” things about the program, areas for program improvement, the online Family Portal, four community-based activities, and overall satisfaction. It also asked if measuring their child’s height and weight had any impact (positive or negative) on their child’s mental health. Interviews and focus groups were completed in person where possible, and otherwise by telephone. See Table 1 for a list of instruments used for data collection at the family level.

### 2.6. Measures: Program Delivery Level

*Adoption, Fidelity, and Barriers and Facilitators to Program Delivery* were determined using a combination of weekly program leader feedback surveys and post-program interviews and focus groups. Adoption was characterised by the proportion and representativeness of providers that deliver the intervention [22]. Fidelity was measured using weekly program leader feedback surveys that had 8 questions (3 multiple choice and 5 open-ended). Two of these questions asked program leaders to check off all the components they were able to complete for that specific week’s session. Also, for each component they were not able to complete, the survey asked them to indicate why (e.g., ran out of time, group was not engaging in the material). Program leaders were also asked if they felt that had the knowledge, support, and ability to successfully deliver that specific week’s content (and if not, why not?). The final question asked if they had any other observations, notes, or feedback about that week’s session. The surveys were completed online after each weekly session using Survey Monkey. An email with the survey link was auto-delivered to each program leader at the end of their weekly session to capture their thoughts, feedback, and experience while it was still top-of-mind.

*Acceptability, Feasibility, Compatibility, and Additional Barriers and Facilitators to Program Delivery* were determined using post-program interviews and focus groups with program leaders, recreation centre staff managers, and the CHLF support team. Interviews and focus groups were semi-structured and included specific questions for each of these roles. Questions were purposely created to ask about key implementation issues including acceptability and barriers and facilitators at both the local and provincial levels. See Table 2 for a list of instruments used for data collection at the program delivery level.

### 2.7. Data Analysis

Qualitative data from post-program interviews was digitally recorded, transcribed using Transcriptive™ software (https://transcriptive.com/wp-admin/setup-config.php), and general categories and themes identified using NVIVO 12. Qualitative data from surveys was exported from Survey Monkey into Microsoft Excel. Once responses were organised, we used a framework analysis approach [27] to identify categories and themes. Themes were then organised based on the process evaluation variables at the family and program delivery levels.

Quantitative data from surveys was exported from Survey Monkey into Microsoft Excel before being analysed descriptively to characterise the sample. Both relative (percentages) and absolute (counts) frequencies were used to provide a clear demographic profile of participants, which aligns with the descriptive objectives of this section.

## 3. Results

Results are divided into two main sections: those at the family level and those at the program delivery level.

### 3.1. Family Level

#### 3.1.1. Reach and Recruitment

In total, 170 children were assessed for eligibility. Of these, 54% (*n* = 92) of the participants heard about the program through social media (Facebook, Instagram, Twitter), 10% (*n* = 17) from posters, 9% (*n* = 15) from media (radio, newspapers), and the rest of 30% (*n* = 51) heard from pediatric physician referral, advertisements from their community recreation centre, or through school newsletter mail outs. Out of the 170 participants assessed for eligibility, 117 children (68%) were invited to participate. The main reasons for exclusion were due to not meeting inclusion criteria (*n* = 24). Inclusion criteria for families to participate in EIP: (1) had at least one child between the ages of 8–12 years old with a BMI ≥85th percentile for age and sex, (2) the child had no known health issues such as cardiovascular disease, mental health issues, or eating disorders, (3) had one parent, caregiver, or legal guardian that was able to attend program sessions with the child, (4) the family resided in British Columbia, and (5) the family was able to commit to the full program schedule. Exclusion criteria included individuals that were unable to participate in physical activity or who were unable to communicate fluently in English.

Overall, out of the 117 children who were invited to participate, 46 children dropped out before commencement (39%) or did not attend the baseline assessment (*n* = 7). The families who chose not to participate due to scheduling conflicts were placed on a list to be contacted for potential participation in future program delivery cycles. Of the 47 children that commenced the EIP, 39 children (83%) and 37 parents completed the program and the process evaluation measures. While there was a comprehensive recruitment effort, participant recruitment as well as participant drop-out before commencement was a challenge; this under-recruitment in family-based interventions is a common and significant issue [28]. Moving forward, this could be addressed by using additional methods in our already multi-faceted approach.

A 2020 systematic review on the “Effective and resource-efficient strategies for recruiting families in physical activity, sedentary behaviour, nutrition, and obesity prevention research” divided recruitment strategies into six themes: school, print/electronic media, community settings, primary-care, employer, and referral-based strategies [28]. The EIP initially used four of these six methods (school, print/electronic media, community settings, and primary care) but did not use an employer- or referral-based strategy. After Program Cycles One and Two, the program added a family-based referral initiative which was communicated to already participating families during their final Week 10 session as well as via email. This method did have some success, although limited. Another method would be to increase recruitment in settings where both children and their parents are present, for example, at school drop-off/pickup, parent nights at schools, and announcements at church events. Guagliano et al., showed that using familiar and trusted relationships is also beneficial, for example, circulating program information from head teachers, family doctors, family friends, and human resources personnel [28].

Overall, the program reached participants with diverse ethnic, educational, and socioeconomic backgrounds enrolling in the EIP (*n* = 47). Specifically, children came from both single-parent and two-parent families; 40% were from non-single parents. Girls and boys participated almost equally in the program (males; 49%). The average age of the participating children was 10.11 ± 1.61 years old. Child ethnicity consisted of 11% (*n* = 5) Indigenous, 40% (*n* = 19) White, 17% (*n* = 8) Asian, 23% (*n* = 11) from multiple ethnicities, and 9% Other (*n* = 4). The most common language spoken at home was English 78% (*n* = 37). A total of 53% (*n* = 25) of the families had a family annual income greater than USD 59,000, and 26% (*n* = 12) parents had completed a bachelor’s or graduate degree. See Table 3 for participants’ demographic characteristics.

#### 3.1.2. Dose Received

The overall completion rate of the EIP program was 83% (*n* = 39 children). Out of those participants who completed the program, 85% children attended 70% or more of the in-person weekly group sessions. Over the 10-week intervention, participating families spent an average of 300 ± 280 min on the Family Portal. Reasons why families withdrew from the program were due to a time conflict (33%), medical reasons or sickness (8%), and lost interest (8%); however, the remaining drop-outs were due to an unknown reason (50%).

#### 3.1.3. Satisfaction, Barriers, and Facilitators to Participation

Families who provided feedback through post-program interviews enjoyed participating in the EIP. Parents who responded were highly satisfied and acknowledged the safe and inclusive program environment. They found the program provided them with the opportunities and tools to make positive changes and were excited about what the program meant for their children. Overall, most parents who completed feedback forms found the information given in the handouts to be easy to understand, respectful, adequate, and suitable for their families and British Columbians in general, with an average rating of 4.4 out of 5. Overall, most parents who completed satisfaction surveys were highly satisfied with how the EIP was delivered, providing an average rating of 4.3 out of 5. A copy of the parent/caregiver satisfaction survey can be found in the Supplementary Materials (S1, page 1).

The areas where parental ratings were lower were in providing families with adequate accurate information before starting the program. Parents felt there were adequate staff members and that staff were knowledgeable and effective. Further, most parents felt the 90-min session was an appropriate length of time and that one session a week for 10 weeks was an appropriate duration. A small number of parents interviewed mentioned that 90 min did not allow enough time.

Overall, most children who completed feedback forms were satisfied with the components of the EIP sessions, with an average rating of 4 out of 5. A copy of the child satisfaction survey can be found in the Supplementary Materials (S2, page 7). On average, children rated 3.4 out of 5 that the individual components helped their family apply what they had learned about living a healthy life and found them useful. Children-only physical activity sessions and family physical activity sessions were the most highly rated components at 4.7 out of 5, respectively. Children were satisfied with the EIP sessions and felt the components helped them apply what they learned about living a healthy life (were useful). Based on the child satisfaction surveys completed after the program, children had fun, liked their program leaders, and plan to make future changes towards living a healthy life with all ratings above 4 out of 5.

Parents identified several barriers and facilitators to participating in or attending the weekly EIP sessions (See Table 4). Predominantly, parents found transportation and scheduling to be the biggest barriers to participating in weekly EIP sessions. Specifically, parents found it challenging to attend sessions that occurred shortly after the workday. Also, parking availability was also identified as a potential barrier to attendance. Participants also identified the length of the program as a potential barrier to attendance (depending on the day of the week) stating that “10 consecutive weeks without a break was also difficult as Sundays is usually the only day where we have time to do things with the family” (parent participant). Participants also identified personal illness and other commitments, such as events around the holidays, as reasons for not attending sessions.

Parents identified several facilitators to participating in or attending the weekly EIP sessions. For example, not having to pay for the program was identified as a facilitator to family participation. The option to enroll multiple children from the same household in the program was also identified as a facilitator to family participants. One participant noted that “allowing siblings to come was definitely the best thing” (parent participant). Parents also appreciated the free family recreation passes and the convenience of the EIP locations and felt that these factors contributed to their attendance of the program.

In the interviews, parents also provided suggestions for improvements. A copy of the parent/caregiver interview questions can be found in the Supplementary Materials (S4, page 13). The key themes were regarding more flexibility as illustrated by one parent who said, “I think options to join by teleconference at times might have been nice. Especially for the follow-up [maintenance] sessions”. Other suggestions for improvement were (1) classes should be longer to allow for more discussion time and more physical activity time and to reduce the amount of homework; (2) extra community-based activities (e.g., grocery store tour) should be scheduled before the program commences so that families know the scheduling commitments well in advance; (3) there should be more program locations so that families do not have to travel as far to attend.

Based on parent and child satisfaction forms completed after the program, families who used the Family Portal consistently liked it and found it useful. Those using the Family Portal also appreciated the ease of use, describing it as “Very easy to use. You don’t need to be computer savvy to use it which is nice. For myself, there were no issues accessing the information or reading any of it, it is concise” (parent participant). Participants also enjoyed the content variety across the Family Portal, specifically having “different kinds of media” and “different things to see”. In general, those who accessed the online resource rated it lower less often.

### 3.2. Program Delivery Level

#### Adoption, Fidelity, Barriers, and Facilitators to Program Delivery

The overall adoption for the EIP was high and British Columbia recreation providers were keen to bring the program to their communities. Several YMCAs and municipal recreation centres responded to a call for expressions of interest in offering the pilot program (*n* = 9). In total, seven of the nine communities originally identified delivered the program. Notably, the EIP was implemented in all five BC regional health authorities: Fraser, Interior, Island, Northern, and Vancouver Coastal. The remaining two sites cancelled the program offering due to low enrollment.

The overall program fidelity was high. A copy of the “Weekly Program Leader Feedback Survey” questions can be found in the Supplementary Materials (S3, page 10). The results showed that program leaders followed the EIP components as they were intended to be delivered most of the time. The average fidelity score for all program components was 73.5% (range 42–95%). The lowest scores were for two positive mental health activities, the appreciation and gratitude circles. The most common reason for not completing components was a shortage of time. This feedback was given in both the weekly feedback surveys and in post-program interviews with program leaders. Also, for appreciation and gratitude circles, leaders often responded that carrying out both activities became repetitive, so combined with a lack of time, they chose to only deliver one of the two circles. “Accomplishments and challenges” had a score of 95% and “tracking and behavioural change” had a score of 78%. Post-program interviews with program leaders and recreation centre staff managers showed acceptability across all sites. Overall, delivery site staff interviewed were keen to be involved in the EIP. Some group leaders described the program as a “natural fit” within their institutions as “the Y [YMCA] is just a family institution and this program kind of goes with those values” (group leader participant). Overall, group leaders described their recreation centre’s response to the program as positive and cooperative. In addition, they felt the participating families formed a community because of their involvement in the program.

Several barriers and facilitators to program implementation were identified by the program leaders (see Table 5). Program leader interview questions can be found in the Supplementary Materials (S5, page 14). For example, small group sizes were identified as a barrier to program implementation as program leaders struggled at times to identify appropriate activities for smaller groups. Also, attendance at the sessions was also identified as a barrier and challenge since it often complicated group dynamics and added to the difficulties of facilitating smaller groups. Group leaders also reported feeling the time constraints of the program, especially when it came to implementing physical activity time, often going over the allotted time to cover the necessary materials. Several facilitators were also identified by the program leaders. Facilitating factors generally fell into three categories: instrumental, educational, and motivational. Instrumental factors generally included room availability for the program and access to equipment. Educational factors included the facilitator’s manual and a training workshop. Finally, motivational facilitators included having experienced and passionate staff and supportive management staff.

Program leaders also identified several areas for improvement including introducing more visual activities for the children to engage with, providing more extensive and detailed training for the program leaders, and more organisation in the teaching materials. Specifically, programs leaders felt that the material could have been made more accessible to the families in a visual format to distance itself from the “textbook” feel that it had. Also, program leaders felt that more pedagogy training would have been beneficial to support their group leadership.

## 4. Discussion

This process evaluation pilot study explored implementation of the EIP in British Colombia, Canada, at both the family and program delivery levels. This study aimed to evaluate reach, recruitment, intervention content, delivery, and implementation of the EIP to enhance quality improvement and inform decisions about potential program scale-up. The EIP aimed to provide an evidence-based program and included several innovative components. For participants, there were positive mental health activities (e.g., gratitude, appreciation, and mindfulness activities), food and physical literacy, screen time, and sleep hygiene activities. For community-based program leaders, there was training on trauma-informed practice, positive mental health, and weight bias. The program also used a more scalable and flexible blended format which introduced families to e-health technologies including the online Family Portal and embedded community resources and activities into the intervention approach. Overall, both the families and program delivery leaders were highly satisfied with the program; it appeared highly acceptable and feasible to implement. However, several participation and implementation barriers and facilitators were identified.

The program reached diverse ethnic, educational, and socioeconomic backgrounds which represented the population demographics of British Columbia, Canada [29]. This included families from all five regional health authorities. Drop-out was comparable to other rates in the literature [30,31]. Reasons why families withdrew from the program included time conflicts, medical reason or sickness, or lost interest, which is consistent with the literature on attrition from childhood weight management programs [31].

The EIP sessions were well attended by those who completed the program. This was comparable to previous studies [32,33,34]. The average contact time was comparable to a systematic review (~26 h) and previous research by Janicke et al. [11]. Families were highly satisfied with both the program content and delivery where most rankings were satisfied or very satisfied. This is comparable to other family interventions where satisfaction ratings were high [32,33,35].

Our qualitative data illustrated some of the potential reasons for program satisfaction. Thematic analysis showed parents acknowledged the safe and inclusive environment provided and felt program leaders were knowledgeable and effective. They reported that the program provided them with the opportunities and tools to make positive changes and they were excited about what the EIP meant for their children. Most children were also satisfied with the program components and felt these components helped them to apply what they learned. They had fun, liked their program leaders, and planned to make future changes towards living a healthy life. “Having fun” was a new factor emerging from our study.

Factors that facilitated participation were that the program was free of cost, siblings were allowed to attend, the program location was convenient, and there was a free family recreation pass. Having a convenient program location is commonly cited in the literature as a facilitator to participation [36,37]. Some families enrolled but were unable to commence because of a program cancellation due to low enrollment. Other barriers to participation included scheduling conflicts, other commitments, transportation challenges, and illness, which is consistent with the literature [36,37,38].

The implementation fidelity for all program components was strong and showed similar fidelity rates to two family-based childhood obesity treatment programs for children ages 5–12 [34]; however, the rate was still lower than a similar program, the MADE4Life program [33]. A contributing factor may be the lack of time to cover all program materials. Based on this feedback from program staff, it was recommended to extend each weekly session from 90 to 120 min. Feedback from program leaders and managers showed acceptability and high compatibility across all sites. Staff were keen to be involved in the EIP. Also, they felt the participating families formed a community as a result of their involvement in the program. All pilot delivery sites indicated they would be interested in continuing to deliver the EIP in their community if funding was made available.

There are several study limitations to consider when interpreting our findings. First, while a comprehensive recruitment effort was undertaken, participant recruitment was a challenge and a limitation to the study. Similarly, a smaller number of families than anticipated participated in the EIP, which we acknowledge limits the generalisability of the study results. Challenges with recruitment and attrition are commonly cited in many other childhood healthy weight interventions [30]. Moving forward, this could be addressed by using additional methods with our already multi-faceted approach, for example, by adding an employer- or referral-based strategy. Another method involves using familiar and trusted relationships such as head teachers and family doctors to circulate program information. Another effective strategy for family-based interventions is to increase recruitment in settings where both children and their parents are present, for example, at school drop-off/pickup, parent nights at schools, and announcements at church events [28].

Another limitation was the completion of satisfaction surveys at the end of program cycles; not all parents and children completed these surveys and most families that withdrew did not complete a survey. In addition, only families that completed the program participated in interviews or focus groups. Based on these limitations, the data is not representative of all families. As in other intervention studies, recruitment bias and selective drop-out may influence the results, and without data from families who dropped out it is difficult to estimate the effect of this. This limitation could be addressed by having a separate short survey for parents and/or children who withdraw from the program, as well as providing them with an opportunity to participate in a short interview to better understand the reasons why they chose to withdraw, in addition to general program feedback and ideas for improvement.

## 5. Conclusions

In summary, the EIP was feasible and highly acceptable for both families and program delivery partners. The program reached diverse ethnic, educational, and socioeconomic backgrounds. The free cost, sibling inclusion, and family recreation passes worked to facilitate the implementation. Recruitment, attendance, online engagement, and limited time to deliver material were challenges that need to be addressed. This feedback was used to recommend changes to the program, for example, extending each weekly session from 90 to 120 min to allow sufficient time to cover all program materials. This study also suggests the benefit of adding an online component to an in-person family-based childhood obesity management intervention which can help to increase program scalability and flexibility.

Future research should focus on strategies to increase recruitment, attendance, and engagement with the online program components. Additionally, it would be beneficial to have a survey for families who withdraw from the program to better understand the reasons why they chose to withdraw. Similarly, individual interviews or focus groups could be used to understand why families withdraw, factors that would help them to successfully complete the intervention, and general program feedback and ideas for improvement. Therefore, this process evaluation study is important for future EIP delivery and scale-up and the use of public funding.

An awareness of the above strengths and limitations can help to inform the development, recruitment, retention, and evaluation of other multi-site childhood healthy weight interventions, as well as the potential for scale-up and subsequent dissemination. It was clear that the resources needed to deliver a free program are a critical scale-up issue as well. Therefore, this study can help to inform decisions regarding the potential design and funding considerations of other family-based childhood obesity management programs.

## Figures and Tables

**Table 1:** Instruments used for data collection at the family level.

Measure	Instrument	Parents	Children	Quantitative/Qualitative
Reach and Recruitment	Demographic and population characteristics (from parent questionnaire)	✓		Quantitative
Dose Received	Attendance forms	✓	✓	Quantitative
	E-session analytics	✓	✓	Quantitative
Satisfaction	Satisfaction surveys	✓	✓	Quantitative and Qualitative
	Post-program interviews and focus groups	✓		Qualitative
Barriers to Participation	Post-program interviews and focus groups	✓	✓	Qualitative
	Satisfaction surveys	✓	✓	Quantitative and Qualitative
Facilitators to Participation	Post-program interviews and focus groups	✓		Qualitative
	Satisfaction surveys	✓	✓	Quantitative

**Table 2:** Instruments used for data collection at the program delivery level.

Measure	Instrument	Program Leaders	Staff Managers	Support Team	Quan./Qual.
Adoption	% of service providers to implement EIP	N/A	N/A	N/A	Quantitative
Fidelity	Weekly feedback surveys	✓			Quantitative and Qualitative
Acceptability	Post-program interviews and focus groups	✓	✓	✓	Qualitative
Feasibility	Post-program interviews and focus groups	✓	✓		Qualitative
Compatibility	Post-program interviews and focus groups	✓	✓	✓	Qualitative
Barriers to Implementation	Post-program interviews and focus groups	✓	✓	✓	Qualitative
	Weekly feedback surveys	✓			Quantitative and Qualitative
Facilitators to Implementation	Post-program interviews and focus groups	✓	✓	✓	Qualitative
	Weekly feedback surveys	✓			Quantitative and Qualitative

**Table 3:** Demographic characteristics (*n* = 47).

Family Demographic Characteristics	*n* (%)
**Languages Spoken at Home**	
English	37 (78%)
Chinese Languages	1 (2%)
Punjabi	3 (6%)
Bilingual	6 (14%)
**Family Income**	
<USD 28,000	16 (34%)
>USD 59,000	25 (53%)
Prefer not to answer	6 (13%)
**Parent Education Level**	
High school diploma	14 (30%)
2-year college	18 (38%)
University	5 (11%)
Graduate degree	7 (15%)
Prefer not to answer	3 (6%)

Note: Asian = South Asian, East Asian, Chinese, and Southeast Asian; Other = Black, Latin American.

**Table 4:** Summary of family barriers and facilitators to participation.

Barrier to Participation	Parent Quotes
Scheduling (*n* = 5)	“It’s a rush to get here in time after work.” “The scheduling was a bit tight for our family and being a Friday night was both good and bad as there tended to be other events especially leading up to Christmas.” “10 consecutive weeks without a break was also difficult as Sundays is usually the only day where we have time to do things with the family.”
Transportation (*n* = 3)	“The traffic on the way to JDF recreation centre was problematic.”
	“Parking spaces at the YMCA.”
No barriers (*n* = 3)	N/A
Illness (*n* = 1)	“Sickness sometimes made it difficult to attend.”
Other response (*n* = 1)	“Life sometimes made it tough, but we made it to as many sessions as possible.”
**Facilitators to Participation**	
Free of cost (*n* = 5)	“No cost to the program to attend helped us a lot.”
	“No cost helped considerably.”
Sibling inclusion (*n* = 4)	“The low barrier access was extremely appreciated, we appreciated that siblings could come.” “Allowing siblings to come was definitely the best thing.”
Location (*n* = 2)	“Location for me was a good one as I lived 10–15 mins away.”
	“Close to home location”
Free family recreation passes (*n* = 2) Other response (1)	“The added gym membership was amazing as well.” “I think options to join by teleconference at times might have been nice. Especially for the follow up [maintenance] sessions.”

**Table 5:** Summary of program delivery barriers and facilitators to implementation.

Barriers to Implementation	Program Leader Quotes
Recruitment	“We need a consistent flow of people getting referred.” “I think the consistency would allow for more involvement from the community as well. And they will be like ‘Okay well this is running twice a year and like, “Oh I’ve heard about it”’.
Small group size	“[in the manual] having more guidelines for us to work on if there’s less participants…having an idea of activities that we can use when we have a smaller group.” “I think because our group was so small, a lot of the activities didn’t work for us or things didn’t look as exact, like, how they were presented. And that wasn’t a huge barrier because we would just substitute. But we didn’t get to do all the activities that you guys wanted us to. Also we had a lot of weeks, well we had one week where nobody showed up.”
Attendance	“Attendance was an ongoing challenge that affected the group dynamic.” “I think attendance at these types of things is always going to be an issue unless there’s some kind of incentive given and total ease of access it’s really accessible”
Limited time to deliver content	“Typically we ended up going over by 15 min every day. So, I think that lengthwise, the budgeted amount of times were a little bit snug for all the activities. I think there needs to be maybe a little bit of time just plugged in for those transitions and things.” “I think the time. We had different start time so only 20–25 min for the physical activity time specifically and I don’t know if we, we can’t do a lot with just 25 min so could that be a little bit longer?”
**Facilitators to Implementation**	
High compatibility and feasibility	“The Y [YMCA] also had like a pool of very qualified, somewhat experienced people especially after the MEND Program. So, it’s again, was kind of a natural fit between having the space and this center having access to all YMCA locations for staffing.”
Room availability	“Having the rooms available and having the set-up, all the rooms— having them ready for us before we start, the rec leaders setting up volleyball for us, things like that.”
Equipment provided	“We have the space. We have the means for it. We have the equipment. I mean I know we were given equipment, but a lot of it… it was just kind of a perfect place to be able to do it if that makes sense.”

## Data Availability

The original contributions presented in this study are included in the article/Supplementary Material. Further inquiries can be directed to the corresponding author.

## Supplementary Materials

The following supporting information can be downloaded at https://www.mdpi.com/article/10.3390/ijerph22101568/s1, S1: Parent/caregiver satisfaction survey; S2: Child satisfaction survey; S3: Weekly program leader feedback survey; S4: Post-program interview questions—parents/caregivers; S5: Post-program interview questions—program leaders; S6: Post-Program Interview Questions—Program Director & Coordinator; S7: Post-Program Facilitator Interview Questions: Support Team.
