# Supplementary material for: The Implementation of a Blended In-Person and Online Family-Based Childhood Obesity Management Program: A Process Evaluation Pilot Study
Source: Int J Environ Res Public Health. 2025 Oct 15;22(10):1568. doi: 10.3390/ijerph22101568 (PMC12562636; doi:10.3390/ijerph22101568)
Supplement: Supplementary file 1 [file ijerph-22-01568-s001.zip › ijerph-3813264-supplementary.pdf]

S1: Parent/Caregiver Satisfaction Survey

This form should be completed by the participating child's **parent or caregiver**.

Thank you very much for participating in the Family Healthy Living Program. We hope you and your child enjoyed it! We want to find out more about what you think of the program so we can make it better for other families like yours. Your feedback is important to make the program a success.

Rating Weekly Sessions
In this section, we want you to think about each session and tell us (1) if you liked it or not, and (2) if you found the session helped you learn and/or you felt it was useful for changing your lifestyle. Please choose a number that corresponds with what you think using a scale of 1 to 5, with 1 being "not at all" and 5 being "a lot" and circle one number per column for each session you attended. Please leave the row blank if you were unable to attend a particular session.
Please leave the row blank if you were unable to attend a particular session.

**Session Name**	**I liked it…**<br>A lot				Not at all	**I learned a lot at this session... It was useful?...**<br>A lot				Not at all
Week 1: Healthy Living Workshop	5	4	3	2	1	5	4	3	2	1
Week 2: Introduction to Healthy Eating and Active Living	5	4	3	2	1	5	4	3	2	1
Week 3: Setting Family Healthy Living SMART Goals and Effective Rewards	5	4	3	2	1	5	4	3	2	1
Week 4: Family Fun at the Supermarket	5	4	3	2	1	5	4	3	2	1
Week 5: Healthy Body Image and Self-Compassion	5	4	3	2	1	5	4	3	2	1
Week 6: Creating Positive and Healthy Family Mealtime & Physical Activity Experiences	5	4	3	2	1	5	4	3	2	1
Week 7: Family, Food and Getting Active Outdoors	5	4	3	2	1	5	4	3	2	1
Week 8: Positive Caregiving and Brainiacs	5	4	3	2	1	5	4	3	2	1
Week 9: Cooking and Playing Together	5	4	3	2	1	5	4	3	2	1
Week 10: Accomplishments & Sticking with it Celebration	5	4	3	2	1	5	4	3	2	1

Rating the Overall Healthy Living Program

In this section, we want know a little bit more about what you think about the Healthy Living Program in general. Using similar scales as before, please tell us (1) if you liked different parts of the Healthy Living Program, and (2) if you found that parts helped your family apply what you have learned about living a healthy life. Please circle one number per column for each part of the Healthy Living Program. **Please leave the row blank if you were unable to attend a particular session.**

Parts of the Healthy Living Program	**Level of satisfaction with…**					**This part helped my family apply what we have learned about living a healthy life… It was useful…**				
	A lot				Not at all	A lot				Not at all
Family Classroom components	5	4	3	2	1	5	4	3	2	1
Child classroom/ Physical Activity components	5	4	3	2	1	5	4	3	2	1
Parent/Caregiver classroom components	5	4	3	2	1	5	4	3	2	1
Family Physical Activity components	5	4	3	2	1	5	4	3	2	1
Handouts for parents and caregivers	5	4	3	2	1	5	4	3	2	1
Handouts for children	5	4	3	2	1	5	4	3	2	1
Online Family Portal	5	4	3	2	1	5	4	3	2	1
Agents of Discovery Game	5	4	3	2	1	5	4	3	2	1
Grocery Store Tour	5	4	3	2	1	5	4	3	2	1
Other additional sessions	5	4	3	2	1	5	4	3	2	1

Rating Components of the Online Family Portal

In this section, we want know a little bit more about what you think about the different components of the Online Family Portal. Using similar scales as before, please tell us (1) if you liked different components of the Online Family Portal, and (2) if you found that components helped your family apply what you have learned about living a healthy life. **Please circle one number per column for each part of the Healthy Living Program. If you did not use the component, please put an "x" under the "Did not use" column.**

**Components of the Online Family Portal**	**Level of satisfaction with…**<br><br>Not at all				A lot	**This part helped my family apply what we have learned about living a healthy life… It was useful…**<br><br>Not at all				A lot	**Did not use**
Articles	1	2	3	4	5	1	2	3	4	5	
Videos	1	2	3	4	5	1	2	3	4	5	
Quizzes	1	2	3	4	5	1	2	3	4	5	
Recipes	1	2	3	4	5	1	2	3	4	5	
Family Activity Ideas	1	2	3	4	5	1	2	3	4	5	
Forum	1	2	3	4	5	1	2	3	4	5	
Our Places	1	2	3	4	5	1	2	3	4	5	
Our Steps	1	2	3	4	5	1	2	3	4	5	

Rating the Information Provided by the Healthy Living Program

In this section, we want to find out more about what you think of the information given in the sessions and the handouts. Please rate the information using a scale of 1 to 5, with 1 being "not at all" and 5 being "definitely."

**Was the information...**	**Information given in sessions**<br><br>Not at all				Definitely	**Information given in the binder**<br><br>Not at all				Definitely
…easy to understand?	1	2	3	4	5	1	2	3	4	5
…culturally suitable for your family?	1	2	3	4	5	1	2	3	4	5
…respectful of your child's issues and constraints?	1	2	3	4	5	1	2	3	4	5
…respectful of your family's financial situation?	1	2	3	4	5	1	2	3	4	5
…enough for you to build a healthy lifestyle?	1	2	3	4	5	1	2	3	4	5
…suitable in your community?	1	2	3	4	5	1	2	3	4	5
…easy to incorporate into your everyday life?	1	2	3	4	5	1	2	3	4	5
…suitable for British Columbians in general?	1	2	3	4	5	1	2	3	4	5

i. Did you learn anything new through the information provided by the Healthy Living Program?

ii. What were the most important pieces of information provided by the Healthy Living Program?

iii. Are there any pieces of information that you think should not be included in the Healthy Living Program in the future?

iv. Were there any areas or topics you expected to be covered that were not?

Rating the Healthy Living Program Delivery

In this section, we want to find out more about what you think of the delivery of the Healthy Living Program. Please rate the program using a scale of 1 to 5, with 1 being "not at all" and 5 being "definitely."

**The Healthy Living Program…**	Not at all				Definitely
…provided your family with enough information before the program started?	1	2	3	4	5
…provided your family with accurate information before the program started?	1	2	3	4	5
…chose an easy to access location?	1	2	3	4	5
…chose a suitable location for the program activities? (rec centre, school, rooms, gym space, etc...)	1	2	3	4	5
…had enough staff members to manage the program effectively?	1	2	3	4	5
…had a knowledgeable Group Facilitator?	1	2	3	4	5
…had an effective Physical Activity Leader?	1	2	3	4	5
…ran the sessions on time?	1	2	3	4	5
…chose an appropriate length for each session (90 minutes)?	1	2	3	4	5
…chose an appropriate frequency for the sessions (1 time per week)?	1	2	3	4	5
…chose an appropriate overall program length (10 weeks)?	1	2	3	4	5

1. Was there anything that made it difficult for your family to attend the Healthy Living Program sessions (e.g. transportation, scheduling)?

2. Was there anything that made it easier for your family to attend the Healthy Living Program sessions (e.g. including siblings in the activities, free program)?

3. What do you think will make it easier for other families to attend the Healthy Living Program in the future (e.g. Providing babysitting)?

4. In your opinion, which part(s) of the Healthy Living Program went well?

5. In your opinion, which part(s) of the Healthy Living Program could use improvement? Please provide details.

Rating the Impacts of the Healthy Living Program

In this section, we want to find out more about the Healthy Living Program's impact on your family.

6. Did your family make any changes towards a healthy lifestyle during the Healthy Living Program?

□ Definitely	□ I think so	□ I don't know	□ I don't think so	□ Definitely not

7. What changes, if any, did your family make?

8. Is your family planning to make any further changes towards a healthy lifestyle after the Healthy Living Program?

□ Definitely	□ I think so	□ I don't know	□ I don't think so	□ Definitely not

9. What further changes, if any, does your family plan to make?

10. What was the one thing you liked the most about the Healthy Living Program?

11. What would you like to change about the Healthy Living Program?

12. Were there any impacts (positive or negative) to your child's positive mental health (eg: social/emotional well-being) from being involved in the Healthy Living Program that you noticed or talked about with your child?

13. Did measuring your child's weight and BMI at the first and last program sessions have any impact (positive or negative) on your child's positive mental health?

14. What would you tell other families about the Healthy Living Program?

15. Would you recommend the Healthy Living Program to other families?

Thank you for your time completing this survey and your participation in the program!

S2: Child Satisfaction Survey

Healthy Living Program Feedback Form – Children

This form should be completed by the **child**.

Thank you very much for participating in the Healthy Living Program. We hope you enjoyed it as much as we did! We want to find out more about what you think of the Healthy Living Program so we can make it better for other children just like you. Your feedback is important to make the Healthy Living Program a success.

Weekly Sessions

First, we want you to think about each session and tell us (1) if you liked it or not, and (2) if you found the session helped you learn about living a healthy life. **Please circle one of the faces in each column to tell us what you think about each session. If you did not attend a session or do not remember a session, please leave the row blank.**

**Session Name**	**I liked it…**<br>A lot … Not at all	**This session helped me learn about living a healthy life… it was useful**<br>A lot … Not at all
Week 1: Healthy Living Workshop		
Week 2: Introduction to Healthy Eating and Active Living		
Week 3: Setting Family Healthy Living SMART Goals and Effective Rewards		
Week 4: Family Fun at the Supermarket		
Week 5: Healthy Body Image and Self-Compassion		
Week 6: Creating Positive and Healthy Family Mealtime & Physical Activity Experiences		
Week 7: Family, Food and Getting Active Outdoors		
Week 8: Positive Caregiving and Brainiacs		
Week 9: Cooking and Playing Together		

Week 10: Accomplishments and Keeping it Going Graduation Celebration		

Program Overall

Now we want to know a little bit more about what you think about the Family Healthy Living Program. Using similar scales as before, please tell us (1) if you liked different parts of the Program, and (2) if you found that part helped you apply what you have learned about living a healthy life. Please circle one face per column for each part of the program. Please leave the row blank if you were unable to attend a particular session.

**Parts of the Healthy Living Program**	**I liked it…**<br><br>A lot Not at all	**This part helped me apply what I have learned about living a healthy life…**<br><br>A lot Not at all
Physical Activity sessions		
Family classroom sessions		
Family Physical Activity components		
Handouts		
Online Family Portal		
Agents of Discovery Game		
Grocery Store Tour		
Other Extra Sessions		

Your Experience in the Healthy Living Program

1. Did you have fun at the Family Healthy Living Program?

A lot Quite a bit It's ok Not really Not at all

2. Did you like your Program Leaders?

A lot Quite a bit They're ok Not really Not at all

3. Did you learn anything new about living a healthy life through participating in the Program?

Definitely I think so I don't know I don't think so Definitely not

4. What was the most important thing, if any, you learned through the Family Healthy Living Program?

5. Did you make any changes in your life so you can be healthier during the Family Healthy Living Program?

Definitely I think so I don't know I don't think so Definitely not

6. What changes, if any, did you make?

7. Do you plan to make any changes in your life so you can be healthier after you finish the Family Healthy Living Program?

Definitely I think so I don't know I don't think so Definitely not

8. What changes, if any, do you plan to make?

9. Do you feel you can live a healthy life after you finish the Family Healthy Living Program? Definitely

I think so I don't know I don't think so Definitely no

10. Do you feel you can live a healthy life after you finish the Family Healthy Living

Program? Definitely I think so I don't know I don't think so Definitely not

11. What was the one thing you liked the most about the Healthy Living Program?

12. What was the one thing you liked the least about the Family Healthy Living Program?

13. Were there any changes in your feelings, positive or negative, (e.g. confidence, happiness) from being involved in the Family Healthy Living Program?

14. What would you tell your friends about the Family Healthy Living Program?

Thank you for your time <u>and participation in the program</u>! :)

S3: Weekly Program Leader Feedback Survey

FHLP Facilitator Feedback Survey - Week 2

Facilitator Feedback

Please complete the following survey based on the **Week 2 session.** This should take approximately 5-8 minutes to complete. Thank you for your feedback and time!

* 1. Facilitator & Session Information

First & Last Name

Date of Session (mm/dd/yyyy)

* 2. Overall, how would you rate this week's session?

- Excellent
- Good
- Fair
- Poor

* 3. How engaged were the children during the physical activity opportunities this week?

- Very engaged
- Somewhat engaged
- Neutral
- Somewhat unengaged
- Very unengaged

* 4. How engaged were the parents/caregivers during the adult-only classroom session this week?

- Very engaged
- Somewhat engaged
- Neutral
- Somewhat unengaged
- Very unengaged

* 5. Please check off all of the session components you were able to complete this week:

Child-only physical activity
Parent/caregiver classroom components:
Family Classroom components
Accomplishments & Challenges
Tracking & Behavioural Change
Family **Action** Break
Appreciation Circle
Gratitude Circle
Activity: Think Before You Drink

For each component that you were not able to complete, please iru:!licate why ,(ran out of time, group was not engaging in the material, etc.)

* 6. Which component(s) from this week's session appeared most effective or well received among **participants?**

Child-only physical activity ....Family Action Break
Parent/caregiver classroom components
Family classroom components
Accomplishments & Challenges
Drink
Tracking & Behavioural Change
Appreciation Circle
Gratitude Circle
Activity: Think Before You

If you have any additional feedback, please enter it here:

* 7. Were there any components or aspects from this week's session that could be improved upon?

Yes

No

If *yes*, which ones? How? Please specify.

* 8. As the facilitator, did you feel that you had the knowledge, supports, and ability to successfully deliver this week's session content?

- [ ] Yes
- [ ] No

If not, why not? Please specify.

* 9. Do you have any other observations / notes / feedback to provide about this session?

S4: Post-Program Interview Questions - Parents/Caregivers

1. What do you think about the Healthy Living program?
2. What impact (positive/negative) has the Healthy Living Program had on your family?
3. Were there any impacts you didn't expect? Either positive or negative? Include spin off benefits.
4. What was the best thing about the program?
5. What was the worst thing about the program?
6. What improvements could be made to the Healthy Living Program to make it more appealing to families in your community?
7. Why do you think that families don't attend? Drop out?
8. What are the major lessons you learned through participating in the Healthy Living Program?
9. To what extent was the program meaningful to you?
10. Was the family web portal useful for you? What did you like? What didn't you like? Is there anything else you would like to see on the portal?
11. What did you think about the extra family activities that were offered?
    - E.g. Grocery store tour
        - What did you like most about this activity?
        - What didn't you like about it, if anything?
    - What did you like most about Agents of Discovery?
        - What didn't you like about it, if anything?
        - Would you recommend it to friends?
12. How satisfied were you with the Healthy Living program?
13. What impact (positive/negative), if any, do you think measuring your child's weight and BMI had on your child's positive mental health? (socially/emotionally)
14. Is there anything else that you feel is important to say

S5: Post-Program Interview Questions - Program Leaders

1. What attracted your recreation centre to participate in the Healthy Living Program?
2. What were your initial reasons for participation in the program? Have your reasons for participation changed since you first started?
3. What has been your recreation centre's response to the Healthy Living Program?
4. What factors do you think facilitated the implementation of the program at your recreation centre?
5. Were there milestones or achievements during the 10-week program that you think are particularly important?
6. Were there barriers to the implementation of the program?
7. Describe your role in implementing the Healthy Living Program pilot.
8. What impact (positive or negative) has the Healthy Living Program had at your recreation centre? In your community?
9. What did you think about the Agents of Discovery application?
    - What worked well/what didn't work well?
    - How did the families respond to the game?
    - Do you think there could be broader use for this type of game within your recreation centre/community?
10. Were there major lessons your recreation centre learned through participating in the program?
11. What do you think of the resources, training, and assistance provided by the Healthy Living Program support team?
12. If funding was announced by the Province, what do you think the likelihood is that your facility would continue to implement the Healthy Living Program?
13. What factors would influence the continued use of the program at your facility?
    - o Prompts if needed:
        - E.g. External incentives for increasing registration and/or encouraging good attendance (7/10 sessions) e.g. grocery store gift card
        - Having a greater (or required minimum) number of participants
        - One of the barriers you mentioned above?
14. What improvements could be made to the program to enhance its chance of success in other recreation centres and communities?
15. What factors do you think would facilitate the provincial roll-out and long-term sustainability of the Healthy Living Program?
16. What factors do you think would be a challenge to the provincial roll-out and long-term sustainability of the program?
17. What have we missed that you feel is important to say?

S6: Post-Program Interview Questions - Program Director & Coordinator

1. What areas do you think worked well/what factors do you think helped with the implementation of the Family Healthy Living Program?
2. What areas/factors do you think were challenges for the implementation of the program?
3. From your perspective, what impact is the program having (positive and negative)? (Implementation)
   Probes
   - On families who participate?
   - On the community in general?
   - Were there any unanticipated benefits to implementing this program?
4. What linkages does the program currently have with other relevant supports and services? Which supports does the program connect with most often?
5. Partnerships: Which partnerships did you draw on for the pilot? (e.g. Shapedown, HealthLINK BC, HEAPK, YMCA, BCRPA, MoH, PHSA, others_What worked well/didn't work well for the partnership Probes
   - Strengths/Weaknesses
   - Barriers/Facilitators
   - Impact
6. What are the conditions for successful longer-term implementation of the Family Healthy Living program at the provincial level?
   Probes
   - Financial sustainability?
   - Relationship/partnership sustainability?
   - Capacity
   - Stakeholder support
7. What aspects of the program's marketing strategy seemed to be effective? What worked well and what could be improved in terms of the combined central/local marketing strategy? Do you have any ideas for how this could be more effective?
8. In your opinion, which part(s) of the support process worked well?
   - Centralized follow-up vs. localized (get the facilitators more involved)
   - Method of communication (phone vs email)
   - Tracking
   - Chain of communication (having you as middle-person between participants and sites)
9. In your opinion, which part(s) of the support process could use improvement?
   - Centralized follow-up vs. localized (get the facilitators more involved)
   - Method of communication (phone vs email)
   - Tracking
   - Chain of communication (having you as middle-person between participants and sites)
10. Are there any major lessons you learned that haven't been raised?
11. Is there anything else that you would like to add?

Additional questions if time permits:

12. To what extent is the Family Healthy Living Program acceptable or meaningful? What changes are needed to increase the program's acceptability or meaningfulness? Can you comment on how this program is/is not filling a gap/need in British Columbia?
13. How does this program meet the needs of different communities/subpopulations? Are there adaptations that need to be made to better meet the needs of residents of British Columbia?

International Journal of
Environmental Research
and Public Health

MDPI

S7: Post-Program Facilitator Interview Questions: Support Team

1. What factors do you think made the program accessible for families to register?
    - E.g.: free, self-referral, convenient location, ability to bring sibling, etc.
2. What, if any, factors did you perceive to be barriers for families registering for the program?
3. How did the BMI requirement impact parents' decision to register for the program? (positive or negative).
    - Did perceived stigma impact decisions to enroll?
4. What were the main reasons that families chose not to register (if their child was eligible)?
5. What do you think will make it easier for other families to register the Healthy Living Program in the future?
6. In your opinion, which part(s) of the recruitment and screening process worked well (from the point of first contact through to Week 1)?
    - Screening script
    - Centralized screening
    - Tracking participant data (including follow-up communication)
7. In your opinion, which part(s) of the recruitment and screening process could use improvement?
    - Screening script
    - Centralized screening
    - Tracking participant data (including follow-up communication)
8. What did you perceive to be the biggest challenge for you to complete a registration?
9. What did you perceive to be the biggest challenge for you throughout the entire registration process from the point of first contact with a family, through to commencement of the program?
10. How did individuals respond to the screening call requirement? (positive or negative)
    10a - Were there any common emotions? (gratefulness for free program, frustration in their inability to effectively help their child, etc.)
    10b - Were there any questions that appeared to illicit negative/strong reactions from individuals?
11. Did you feel comfortable/knowledgeable referring parents/caregivers to external resources when needed?
12. Is there anything we missed that you feel is important to say?
